# Evaluation of the Effectiveness of Laser-Assisted Bleaching of the Teeth Discolored due to Regenerative Endodontic Treatment

**DOI:** 10.1155/2022/3589609

**Published:** 2022-07-06

**Authors:** Noushin Shokouhinejad, Mehrfam Khoshkhounejad, Fatemeh Hamidzadeh

**Affiliations:** ^1^Department of Endodontics, School of Dentistry, Tehran University of Medical Sciences, Tehran, Iran; ^2^Dental Research Center, Dentistry Research Institute, Tehran University of Medical Sciences, Tehran, Iran

## Abstract

Regenerative endodontic treatments (RETs) as a valuable treatment option to save the immature necrotic teeth, have been reported to be associated with discoloration which is an inevitable unfavorable outcome. The present study aimed to compare three laser-assisted protocols with conventional walking bleaching in terms of bleaching efficacy. Seventy-two human incisor teeth underwent regenerative treatment. A triple antibiotic paste containing minocycline, ciprofloxacin, and metronidazole was used as an intracanal medicament. A human blood clot was applied as a scaffold and capped by a hydraulic calcium silicate-based cement. Ten weeks after the RET procedure, a four-session bleaching course started. Teeth were assigned to four groups: (1) 35% hydrogen peroxide gel, (2) 35% hydrogen peroxide gel + Nd: YAG laser, (3) 35% hydrogen peroxide gel + 980 nm diode laser, and (4) 35% hydrogen peroxide gel + 810 nm diode laser. The color changes (ΔE) were measured before and after bleaching sessions. The data were analyzed using Kruskal–Wallis nonparametric test. The statistical significance level was set at 0.05. Significant discoloration, exceeding the perceptibility threshold (ΔE > 3.7) was observed in all of the samples ten weeks after RET. There was no significant difference between groups in terms of RET-induced discoloration values (*p* > 0.05). Bleaching either by using 35% hydrogen peroxide or 35% hydrogen peroxide activated by different lasers used in this study resulted in significant tooth whitening (*p* < 0.05). There was no statistically significant difference among the groups in terms of bleaching efficacy (*p* > 0.05). Internal bleaching by using 35% hydrogen peroxide is as effective as laser-assisted protocols for correction of crown discoloration in teeth that have undergone RET.

## 1. Introduction

Regenerative endodontic treatments (RETs) are primarily based on the knowledge that dentin-pulp complexes would be regenerated in a bacteria-free root canal in the presence of an appropriate scaffold, stem cells, and essential growth factors [1]. RETs are assumed to be superior to apexification because they provide continuous root development and resulting in a higher resistance to fracture [2]. The main steps of RET are root canal disinfection, application of an appropriate scaffold, and providing a biocompatible hermetic coronal seal [3].

RETs' outcomes are mainly categorized as patient-, clinician-, and scientist-centered outcomes. Patient-centered outcomes include resolution of disease, tooth survival and function, and favorable esthetics. Although successful radiographic and clinical outcomes are found in most available publications, coronal tooth discoloration has been commonly reported in several studies following these treatments. Thus, tooth discoloration still remains a nonnegligible unfavorable outcome especially while considering these treatments for anterior teeth [4]. Several factors may affect tooth discoloration induced by RETs, including the intracanal medicaments especially those containing tetracycline derivative medications used for canal disinfection; the presence of blood clot as a scaffold; and the application of hydraulic calcium silicate-based cements (HCSCs) as an intracanal coronal barrier at the level of cemento-enamel junction (CEJ) especially in contact with blood clot [5–8].

Although there is enough evidence indicating the incidence of discoloration following RETs, limited information is currently available on the most effective management of this unfavorable outcome. Tooth discoloration could be eliminated by using a veneer or crown. However, these procedures are invasive and should not be considered as the first choice for treatment. It seems tooth bleaching procedures are more reasonable as they are viable, simple, and less invasive [9]. Several *in vitro* studies have assessed the efficacy of internal bleaching in teeth discolored by different antibiotic pastes [10–14] and found this procedure to be quite effective in the correction of tooth discoloration. In addition, internal bleaching has been evaluated for the whitening of teeth discolored due to RETs in some case reports and case series which resulted in satisfying outcomes in most of the cases [15–19].

Thermo/photo-bleaching uses light as a facilitator in internal bleaching. LED and KTP laser-assisted internal bleaching have been shown to be beneficial in the management of tooth discoloration induced by tetracycline, which is known to be resistant to treatment [20]. Furthermore, it has been suggested that diode lasers are more effective and less time consuming in comparison with LED lasers in external bleaching [21]. There are few studies comparing the effectiveness of laser in the bleaching of discolored teeth caused by the mixture of antibiotics used in RET [10]. To the best of our knowledge, to date, no previous studies have evaluated the effectiveness of laser-assisted bleaching of discolored teeth after the completion of RETs. It should be noted that the final clinical appearance in teeth undergoing RETs, is the consequence of multiple discoloration-inducing factors interacting in a complex biologic environment. Thus, to have more reliable data, the effectiveness of internal bleaching techniques must be evaluated in a model while all the potentially discoloring agents are present in order to simulate the clinical situation. Therefore, the present study aimed to evaluate and compare the effectiveness of three laser-assisted (Nd: YAG, 980 nm and 810 nm Diode) lasers to the conventional internal bleaching technique for correction of tooth discoloration induced by RET.

## 2. Materials and Methods

Seventy-two human incisor teeth which were caries- and restoration-free and extracted due to either orthodontic or periodontal reasons, were included in the present *ex-vivo* study. Teeth were disinfected in chloramine-T 0.5% for 48 hours and then stored in normal saline. After taking periapical radiographs, teeth with any signs of cracks, resorption, immature apices, and caries/restorations were excluded. Then the apical part of all roots was cut in order to maintain only 10 mm of the root below the buccal CEJ. After access cavity preparation, root canals were prepared using #1 to #5 gate-glidden burs. The apical terminus of the canal was sealed by resin modified glass ionomer (Masterdent, Dentonics, USA). The subsequent steps of the regeneration protocol were done according to AAE clinical considerations for a regenerative procedure [22]. Canals were irrigated with 20 mL of NaOCl 1.5% and 20 mL of EDTA 17%. Access cavity walls were covered by a dentin bonding agent (3M Adper Single Bond, 3M, USA). A triple antibiotic paste (TAP) composed of ciprofloxacin, metronidazole, and minocycline (1 : 1 : 1) mixed with distilled water in 1 mg/mL concentration was prepared. The antibiotic mixture was applied in the root canals just below the CEJ. The access cavity was sealed by a temporary restoration material (Zonalin, Kemdent, London, UK). Teeth were incubated at 37°C in fully saturated humidity for 4 weeks. After that, the access cavities were reopened and the canals were irrigated with 20 mL EDTA 17% and dried with paper points. The root canals were filled with human blood using a syringe up to 3 mm below the facial CEJ and allowed to form a blood clot for 15 minutes. The whole fresh human blood used in this study was collected from healthy consented volunteers and approved by the Tehran University of Medical Sciences Ethical Committee (IR.TUMS.MEDICINE.REC.1400.123).

Then a 3 mm-thick coronal barrier of RetroMTA (BioMTA, Korea, Seoul) prepared according to the manufacturer's instructions was applied over the blood clot. A moistened cotton pellet was inserted over the RetroMTA. The cavity was temporarily sealed with Coltosol (Coltene, Altstätten, Switzerland). The specimens were incubated for 24 hours. Subsequently, after checking the setting of RetroMTA, the access cavities were filled with composite resin material. Afterwards, the teeth were stored at 37°C with fully saturated humidity for 10 weeks.

After 10 weeks, the composite resin was removed. To ensure the removal of dentin bonding agent from the dentinal tubules, access cavity walls were slightly freshened by a high-speed diamond fissure bur. Teeth were randomly assigned to four groups. In order to prevent confounding bias, the samples were stratified based on their RET-induced discoloration (measured in Δ*E*). Stratification was conducted in a way that Δ*E* range of the teeth in each stratum did not exceed 4 units. We had nine strata, each containing eight samples which were assumed to be blocks. In each block, samples were randomly assigned to four groups in a 1 : 1 : 1 : 1 ratio.

The bleaching procedure was performed in four groups as follows (*n* = 18):

  35% hydrogen peroxide (H_2_O_2_): The pulp chamber was filled with H_2_O_2_ gel (Opalescence®Endo; Ultradent Products Inc., USA)
  35% H_2_O_2_ + Nd: YAG laser (Power: 2.5 W, Frequency: 25 Hz)
  35% H_2_O_2_ + 980 nm Diode laser (Power: 2 W, Mode: Continuous wave)
  35% H_2_O_2_ + 810 nm Diode laser (Power: 2 W, Mode: Continuous wave)

In the laser-assisted groups, 35% H_2_O_2_ gel was applied in the pulp chamber, followed by laser radiation for 30 seconds three times with one-minute intervals. In all groups, H_2_O_2_ gel was left in the pulp chamber till the next session of bleaching, and the access cavity was temporarily filled with Coltosol (Coltene, Altstätten, Switzerland). The bleaching agent (35% H_2_O_2_) was replaced and the bleaching process was repeated at days 4, 8, and 12.

## 3. Tooth Color Assessment

The color of the crown was measured by spectrophotometry (VITA Easyshade V; Zahnfabrik, Bad Säckingen, Germany). The color assessment was performed by the same investigator under steady laboratory conditions by one calibrated operator two hours after the bleaching procedure. The device was calibrated before being used for each specimen. The spectrophotometer was used to measure the L^*∗*^, a^*∗*^, and b^*∗*^ values. L^*∗*^ indicates the value of lightness-darkness, a^*∗*^ indicates greenness-redness, and b^*∗*^ indicates blueness-yellowness.

Color measurement was done 2 mm above the center of the CEJ of the labial surface and performed at six different time periods:

  T0 (before RET): baseline or before regenerative endodontic treatment
  T1 (after RET): ten weeks after regenerative endodontic treatment
  T2 (1^st^ bleaching session): after performing the first bleaching procedure
  T3 (2^nd^ bleaching session): after bleaching on the day 4
  T4 (3^rd^ bleaching session): after bleaching on the day 8
  T5 (4^th^ bleaching session): after bleaching on the day 12

At each time interval, the color measurements were repeated three times. The mean value of three measurements was calculated and recorded. The color change (Δ*E*) between each time interval was calculated using the following formula:

(1) $$\begin{array}{c} \Delta E={\left[{\left(\Delta L\right)}^{2}+{\left(\Delta a\right)}^{2}+{\left(\Delta b\right)}^{2}\right]}^{1/2}. \end{array}$$

Statistical analyses were performed using SPSS 26.0 software (SPSS Inc., Chicago, IL). All statistical analyses were performed at a significance level of 0.05 and confidence interval of 95%. Δ*E* values of the experimental groups were compared in each time period. The groups were compared for differences in mean Δ*E* value at different time intervals using Kruskal–Wallis nonparametric test. To ascertain which differences were clinically visible, the human perceptibility threshold was set to 3.7 units (Δ*E* > 3.7) [23, 24].

## 4. Results

**Figure 1:**
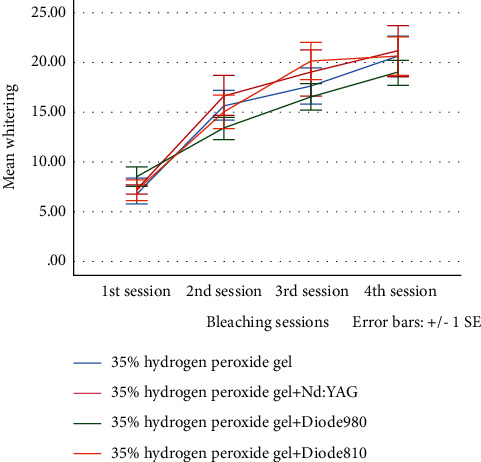
Mean whitening measures after each bleaching session.

Discoloration was observed visually in all specimens ten weeks after RET and all of the experimental groups showed color changes exceeding the perceptibility threshold (Δ*E* > 3.7) (Figure 1). The mean values and standard deviation of the effect of each bleaching procedure at different time intervals are shown in Table 1.

The results showed that there was no significant difference between the bleaching techniques used in this study (*p* > 0.05).

In the intragroup analysis, the findings of the current study indicated significant color correction at the 1^st^, 2^nd^, and 4^th^ bleaching sessions in each group (*p* < 0.05). However, bleaching using 35% H_2_O_2_ + 810 nm Diode laser was also associated with significant tooth whitening at the 3^rd^ session of bleaching (*p*=0.012).

**Figure 2:**
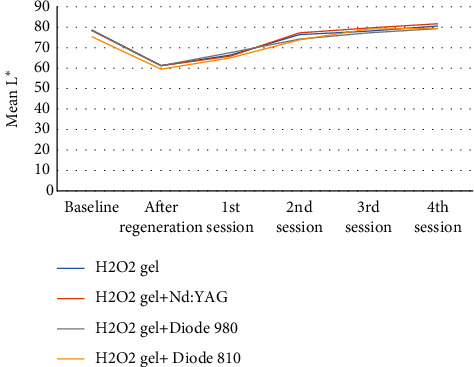
L^*∗*^ mean changing trends in each group during the study.

Considering the L^*∗*^ values, the results suggested an increase over the subsequent time periods in each group. After the 4^th^ session of bleaching in all groups, L^*∗*^ values significantly increased compared to after-RET L^*∗*^ values (*p* < 0.001) reaching the baseline values (Figure 2).

**Figure 3:**
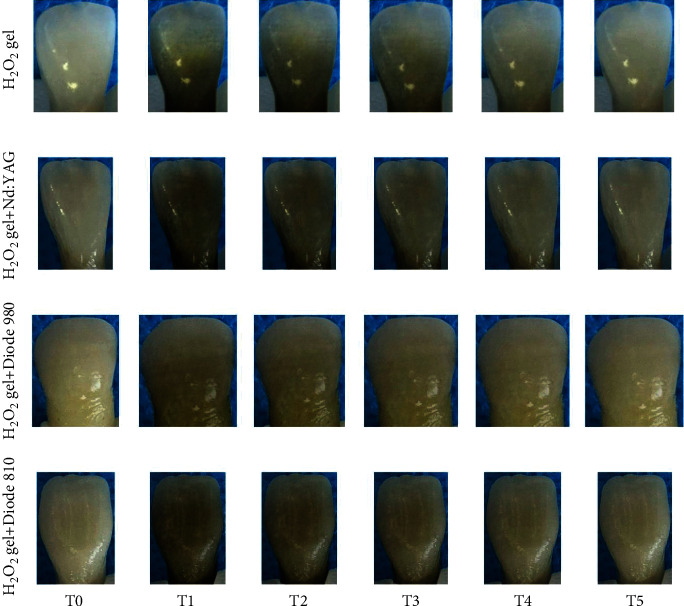
Representative images of the specimens in each experimental group during the study.

Photos of a specimen from each group are shown in Figure 3.

## 5. Discussion

The present study aimed to make a comparison between photo-bleaching protocols using different lasers and conventional internal bleaching techniques in the correction of tooth discoloration induced by RET.

In this study, all specimens revealed a clinically perceptible discoloration after RET (Δ*E* ≥ 3.7). This is in agreement with previous studies showing considerable crown discoloration in teeth that have undergone RET-related procedures [8, 25, 26]. In the present study, a low concentration of TAP containing minocycline was used for 4 weeks as an intracanal medicament, according to the last version of the American Association of Endodontists (AAE) clinical considerations for a regenerative procedure. A considerable contribution of minocycline-containing TAP to the crown discoloration has been shown even after the sealing of the pulp chamber dentinal tubules with an adhesive dentin bonding agent [26]. The application of blood clots as scaffolds is considered another source of crown discoloration [27], possibly due to the penetration of red blood cells into the dentin and accumulation of iron-containing products in the dentinal tubules [6, 28]. In RETs, the proximity of the blood clot scaffold to the CEJ might be a reason for discoloration of the cervical third of the crown. Furthermore, interaction of blood with the unset HCSCs, which are located in the most coronal part of the root might be another reason for discoloration of HCSCs when exposed to blood. It has been shown that blood contamination exacerbated discoloration induced by HCSCs regardless of the type of radiopacifier in the composition [6–8, 29]. During a natural redox reaction, ferrous (Fe^2+^) ions in the heme group of blood which possesses a red color become ferric (Fe^3+^) which has a dark brown color that might result in discoloration of HCSCs [6–8, 29].

In the current study, the CIE L^*∗*^ a^*∗*^ b^*∗*^ color system (International Commission on Illumination) by means of spectrophotometry was used to measure discoloration of teeth after RET and also correct discoloration using different bleaching techniques. There are several means to measure tooth color, e.g., shade guides, spectrophotometers, colorimeters, spectroradiometers, and digital camera and imaging systems [30]. Spectrophotometry is based on the measurement of the amount of light energy reflected from an object at 1–25 nm intervals along the visible spectrum [31]. It has been suggested as a repeatable and accurate method of color measurement [30].

The present study showed no significant difference between photo-bleaching by different lasers and conventional internal bleaching using H_2_O_2_, corroborating previous study's findings revealing no difference between photo-bleaching by Nd: YAG and walking bleaching using 35% hydrogen peroxide gel in teeth treated with different antibiotic pastes [10].

In the current study, the bleaching effect on the 12^th^ day was significantly higher than the other days which is in agreement with previous studies [10, 14] showing that bleaching effects on the 12^th^ day were superior to earlier sessions of bleaching.

Based on spectrophotometric findings, higher L^*∗*^ values and lower a^*∗*^ and b^*∗*^ values are associated with more favorable bleaching outcomes. In other words, either an increase in lightness or reduction in redness or yellowness means whitening in the CIE L^*∗*^a^*∗*^b^*∗*^ system [32]. In the present study, the approximate baseline luminosity values (L^*∗*^ values) were obtained at the end of the bleaching course in all groups. The same trend of L^*∗*^ changes was observed by Akbulut et al. [25] who compared color correction of discolored teeth undergone RETs with the application of different calcium silicate-based cements. Furthermore, Kirchhoff et al. [12] showed a significant increase in L^*∗*^ reaching the baseline values after internal bleaching of teeth discolored due to the application of TAP as an intracanal medicament.

Minocycline, as a part of TAP used as an intracanal medicament, has a significant contribution to tooth discoloration [15, 26, 33, 34]. Several studies have proved the efficacy of intracanal antibiotic compositions other than TAP with minocycline, such as DAP, and TAP containing cefaclor or clindamycin [33, 35]. The other option suggested by the European Society of Endodontology is calcium hydroxide [36]. Considering aesthetics as a patient-oriented outcome, using alternative intracanal medicaments might lead to a more successful RET. Although in the present study, according to AAE clinical considerations for a regenerative procedure, TAP with minocycline was applied as an intracanal medicament, it seems rational to substitute this medicament with the alternatives in RET protocols in order to achieve more favorable results.

According to the literature most of the cases have been treated by recruiting blood clots formed following the induction of bleeding from the periapical area [37]. Considering the discoloration potential of blood per se and the role of it in intensifying the discoloration caused by HCSCs [5], alternative scaffolds may be preferred in RETs, provided that they meet the criteria of an ideal scaffold. Scaffolds such as platelet-rich fibrin (PRF) and platelet-rich plasma (PRP) have been associated with favorable and successful results in previous studies [38–43]. Although PRF has been addressed as an ideal purely autologous scaffold for regeneration [44, 45], there is not enough evidence available to substitute blood clot with another scaffold.

In the present study, according to AAE clinical considerations for a regenerative procedure, RetroMTA was used as the coronal barrier material. This cement contains a calcium-zirconia complex as a radiopacifier. Calcium silicate-based materials used as coronal barrier materials in RETs are another source of discoloration. It has been suggested in previous studies that bismuth oxide-containing HCSCs have more discoloration potential [46]. Thus, zirconium oxide- or tantalum oxide-containing HCSCs with significantly less discoloration potential [5, 47, 48], are appreciated in RET protocols.

Aging and periodontal disease are among the factors that significantly contribute to tubular sclerosis [49, 50]. As the teeth recruited in the present study were mature teeth and were mostly extracted due to periodontal problems, they might not be suitable representatives of immature teeth. It means that implementing the same RET protocol in the clinical settings may lead to a more severe discoloration because of the greater number and size of dentinal tubules; thus, the management of such discoloration might be more challenging. It means that, along with attempts to establish efficient guidelines to better manage RET-induced discoloration, clinicians must try to minimize discoloration through possible modifications in clinical procedure.

Hydrogen peroxide 35% is a potentially caustic bleaching agent. It should be used with caution, especially when an immature tooth is the case [14, 51]. In such cases, it is suggested to use lower concentrations of hydrogen peroxide or less-effective-but-safer bleaching agents such as sodium perborate [52]. Although it is not contraindicated to use 35% hydrogen peroxide over a thick-enough coronal barrier for treatment purposes even in immature teeth [52], a conservative clinician may decide to use a less potent bleaching agent. Laser-assisted bleaching outcomes with a less potent bleaching agent should be investigated in future studies.

## 6. Conclusions

Within the limitations of the present study, management of the discoloration following RETs would be possible with either conventional walking bleaching or laser-assisted bleaching. Activation of 35% hydrogen peroxide with different lasers, i.e., Nd: YAG, 810 nm and 980 nm diode did not lead to a significant difference in the bleaching outcomes.

## Figures and Tables

**Table 1:** Mean whitening values and standard deviations for each group at each bleaching session.

Group	Bleaching session			
	First (T2)	Second (T3)	Third (T4)	Forth (T5)
H_2_O_2_ gel	6.78 ± 4.35	15.65 ± 6.44	17.62 ± 7.64	20.61 ± 8.62
H_2_O_2_ gel + Nd: YAG	7.26 ± 2.01	16.69 ± 8.36	18.94 ± 9.79	21.17 ± 10.71
H_2_O_2_ gel + 980 nm diode	8.55 ± 4.10	13.39 ± 4.86	16.55 ± 5.71	18.94 ± 5.33
H_2_O_2_ gel + 810 nm diode	7.16 ± 4.40	15.04 ± 7.19	20.18 ± 7.87	20.66 ± 8.12

## Data Availability

The data supporting the findings of the present study are available from corresponding author upon request.
